# The Use of Complementary and Alternative Medicine among Korean Young Adult Members of Fitness Centers

**DOI:** 10.1155/2019/7648237

**Published:** 2019-02-24

**Authors:** Seung-Nam Kim, Bokmin Kim, Jaehee Kim

**Affiliations:** ^1^College of Korean Medicine, Dongguk University, Goyang-si, Gyeonggi-do, Republic of Korea; ^2^Graduate School of Alternative Medicine, Kyonggi University, Seoul, Republic of Korea

## Abstract

In general, the pattern and perception of complementary and alternative medicine (CAM) use in young people are little known. Particularly, given South Korea's dual health care system that includes both Korean traditional medicine and Western medicine, young adults in South Korea may be unique for the study of CAM use. Accordingly, this study investigated the modality, purpose, and perceptions of CAM use among young adults in South Korea and determined the predictors of CAM use. In addition, reasons for CAM use were compared to those for exercise. A survey was conducted among 649 young members of fitness centers (aged 20-39 years). The structured written questionnaire included the questions related to use of 30 CAM modalities, satisfaction with CAM use, factors associated with CAM use, reasons for exercise and CAM use, and perceptions of CAM. The most common therapies used in lifetime were acupuncture, massage, moxibustion/cupping, yoga, and diet-based therapies. The most satisfied therapy was massage followed by aroma therapy. The main reasons for using CAM were to relieve musculoskeletal pains while those for doing exercise were to lose weight, to promote health, and to have a positive body image. Multiple logistic regression analysis revealed that higher education level, having a religion, and having a health problem were significant independent predictors of CAM use after controlling for other factors. The majority of respondents reported “relief of pain and symptoms” as the perceived effect of CAM and “lack of advertising” as the weakness of CAM. The majority of respondents got CAM information from mass media and Internet. In conclusion, CAM use is significantly associated with education level, religious status, and health status in Korean young adult members of fitness centers. The main purposes of utilizing CAM and performing exercise are different.

## 1. Introduction

Prevalence of complementary and alternative medicine (CAM) use is different across countries depending on diverse cultural and social backgrounds [1, 2]. CAM use is associated with several factors such as sex, age, education, and income [1, 3]. In general, it is well-known that CAM use increases with age [1–3], but few studies have examined CAM use in younger adults: Most of previous studies about CAM use have been focused on users in older age since CAM users tended to be middle-aged or elderly [1, 3–5]. Previous studies about CAM use in young people were mostly focused on university students [6–10] and their use of dietary supplements (17%-65%).

Unlike most countries, Korean traditional medicine is recognized as a conventional medicine in the national healthcare system of South Korea, but a separated medical system with Western medicine [11]. In addition, the overall reputation of Korean traditional medicine in South Korea has rapidly improved in the 1990s. Therefore, the patterns and perceptions of CAM use in young adults in South Korea may be different from older adults and those in other countries.

Since it is generally not known about the patterns, reasons, and perspectives of CAM use in young adults, we investigated these aspects of CAM use in young adults. Moreover, we also investigated whether reasons for CAM use and exercise are different in young adults, especially among physically active young adults, because CAM and exercise are common nonpharmacological and nonsurgical approaches for health promotion and the relief of diseases and symptoms [12–14]. Generally, young adults are known to engage in exercise more than older ones [14] and exercise is associated with other health-related behaviors in young adults [15]. Fitness center members exercised more regularly and were more physically active and less sedentary [16, 17]. Given the similarity of exercise and CAM as preventive health care modalities and the age-related differences in utilization of both modalities, we investigated the prevalence, modalities, predictors, reasons, and the perceived benefits and weaknesses of CAM use among young adult members of fitness centers and determined the factors related to CAM use. In addition, reasons for CAM use were compared to those for exercise.

## 2. Methods

### 2.1. Study Design

The survey was conducted at 7 local commercial fitness centers in Seoul and Gyeonggido in South Korea in 2016. Ethical approval for this study was obtained from Institutional Review Board of Korea National Institute for Bioethics Policy (IRB approval No. P01-201602-23-002). The IRB approved that the study protocols met the criteria for IRB exemption and did not require individual informed consent. All participation was voluntary and anonymous.

### 2.2. Study Sample

The survey was conducted among 720 young adults (aged 20-39 years) enrolled in fitness centers at the time of study participation. The majority of respondents were doing aerobic and resistance exercises. Seventy-one subjects with incomplete survey were excluded. A total of 649 young adults completed the survey.

All participants were asked to anonymously and voluntarily complete the structured written questionnaire consisted of a series of closed-ended questions. The questionnaire was administered by a staff member at the fitness center. The members of fitness centers were asked if they would be willing to participate in a survey of CAM use. Among them 720 respondents had agreed to participate in the survey.

### 2.3. Measures

#### 2.3.1. CAM Use

In this study, the following 30 types of CAM modalities were included to define CAM use: acupuncture, moxibustion and cupping, Ayurveda, homeopathic treatment, ginseng, herbs (except ginseng), other natural products (probiotics, enzyme, etc.), diet-based therapies, vitamin and mineral supplements, aroma therapy, folk remedies (animal extracts, etc.), massage and acupressure, spinal manipulation (chiropractic or osteopathic manipulation), Chuna (manipulative therapy practiced in Korean traditional medicine), pilates, yoga, meditation, tai chi, Kouksundo (Korean mind-body practice), art therapy, hypnosis, progressive relaxation, guided imagery, qi gong, prayer therapy, magnetic therapy, light therapy, taping therapy, therapeutic spa in hot springs, and hand acupuncture.

Our list of CAM modalities was partially based on the US National Health Interview Survey [1, 18–20] and was further augmented with a list of modalities from a previous study of CAM use in Korea [18]. This enabled us to include the modalities commonly used in South Korea, which may not be common in other parts of the world.

Participants were asked for their lifetime experience with CAM use and what CAM modality they used. Respondents who have ever used any of 30 modalities of CAM were defined as CAM users. Respondents who used none of the 30 modalities of CAM were considered as non-CAM users. User's satisfaction with CAM was also assessed. Satisfaction levels of each CAM therapy were measured by a 5-point Likert scale (1=very dissatisfied; 2=somewhat dissatisfied; 3= neutral; 4= somewhat satisfied; 5= very satisfied) [21, 22].

#### 2.3.2. Factors Associated with CAM Use

The variables, which were used to examine the factors associated with CAM use, included sociodemographic characteristics, perceived general health status (very good, good, fair, poor, or very poor), and presence of health problem [1–3, 19, 20, 23]. Sociodemographic characteristics were as follows: age groups (20–29 years or 30–39 years), sex, education (less than high school, high school, or college or higher), marital status (single, married, or others), religious status (none or having a religion), employment status (unemployed or employed) and monthly family income. Income was divided into <2 million Korean Won (KRW), 2-4 million KRW (about US$1800-$3600), 4-6 million KRW, or ≥6 million KRW ($5400).

Because there were no respondents who answered ‘others' to the question of marital status, it was excluded from the data analysis. Only 9 respondents had less than a high school diploma. The education level was recategorized into “high school or less” or “college or higher”. Presence of health problem was defined as presence of at least one physical illnesses or mental illness among the following lists: hypertension, hyperlipidemia, heart diseases, stroke, diabetes, stomach disease, renal disease, lung/respiratory diseases, cancer, neck/shoulder pain, headache, back pain, sleep disorder, depression/anxiety/emotional problem, arthritis/rheumatism, osteoporosis, liver disease, and others [4, 14, 19, 20].

#### 2.3.3. Reasons for Exercise and CAM Use

Reasons for CAM use and adverse events experienced with CAM use were asked. In addition, reasons for doing exercise were asked to all respondents.

#### 2.3.4. Perceptions of CAM

The questionnaires related to perceptions of CAM included the perceived benefits of CAM, the weaknesses of CAM, and the CAM information resources. The respondents were asked the following questions: “What do you think about the benefits of CAM?”; “What do you think about the weaknesses of CAM?”; “Where do you get the information about CAM?”. The multiple-choice questionnaires were used and the response choices were presented as categories of variables in Table 5.

### 2.4. Statistical Analysis

Chi-square analyses and unadjusted logistic regression analyses were used to explore the associations of sociodemographic and health-related factors with CAM use. In addition, a multiple logistic regression analysis was performed to identify independent predictors statistically significantly associated with CAM use, while controlling for other variables in the model. Both unadjusted and adjusted Odd ratios (ORs) of CAM use were calculated with 95% confidence intervals (CIs). Data were analyzed using SPSS version 22.0 (IBM SPSS Inc., Chicago, IL). The level of significance was set at *α* = 0.05.

## 3. Results

### 3.1. Sociodemographic and Health-Related Characteristics

Of 649 respondents, 55% were aged 20-29 years, 56% female, 88% single, 74% college graduate or higher, 77% employed, and 68% not having a religion (Table 1). Overall, 85% of respondents perceived their health as fair or good. About 43% of respondents had at least one health problem. Of those with health problem, 31% had the neck and shoulder pain, 11% headache, and 11% back pain (Table 1).

### 3.2. Prevalence and Predictors of CAM Use

Overall, 81% of respondents had used at least one of 30 CAM therapies in lifetime (Table 2). As presented in Table 1, users of CAM were significantly more likely to be older compared with nonusers (47% versus 37%), female (57% versus 47%), married (13% versus 6.5%), and employed (79% versus 70%). They were significantly more likely to have a higher education (77% versus 63%), a religion (34% versus 22%), and health problems (47% versus 24%). Monthly family income and self-perceived health status were not related to CAM use. Findings of unadjusted logistic regression analysis (Table 3) were similar to those of chi-square analyses.

Results of multiple logistic regression analysis (Table 3) revealed 3 significant independent predictors of CAM use. Higher education (adjusted OR, 1.76; 95% CI, 1.11–2.77), having a religion (adjusted OR, 1.75; 95% CI, 1.08–2.83), and presence of health problem (adjusted OR, 2.79; 95% CI, 1.72–4.54) were significantly associated with CAM use after controlling for age, sex, marital status, employment status, income, and self-perceived health status.

### 3.3. Use of CAM Modalities and Satisfaction with CAM

The 10 modalities reported as most frequently used were acupuncture; massage; moxibustion/cupping; yoga; diet-based therapies; pilates; ginseng; taping therapy; aroma therapy; and other natural products (Table 2). The most satisfied therapy with CAM users was massage, followed by aroma therapy, Chuna, spinal manipulation, taping therapy, yoga, pilates, other natural products, moxibustion and cupping, and acupuncture (Table 2). Among CAM users, 41.8% used 1-3 different CAM therapies; 34.6% used 4-6 therapies; 23.6% used 7 or more therapies. The average number of therapies used was 4.8 ±3.5.

### 3.4. Reasons for CAM Use and Exercise

The most frequently reported reason for CAM use was for neck and shoulder pain (32.5%), followed by back pain (12.2%), chronic fatigue (7.7%), stress management (7.7%), arthritis (5.7%), disease prevention and health promotion (5.1%), and obesity management (4.6%) (Table 4). Only 3 persons among 271 respondents reported to experience adverse events with CAM therapies. Reasons for doing exercise were “weight loss” (62.9%), “health promotion” (53.8%), “to have a positive body image” (44.8%), “stress management” (25.1%), “leisure activity” (18.6%), “relief of pain” (8.6%), “treatment of disease” (4.3%), and “others” (0.8%).

### 3.5. Perceptions of CAM

The benefit and weakness of CAM and the CAM information resources are presented in Table 5. About 58% of respondents perceived “relief of pain and symptoms” as the benefit of CAM. Other perceived benefits were “psychological and emotional wellbeing” (40.1%), “promotion of health and physical fitness” (33.1%), “improvement of immune function” (12.2%), and “treatment of disease” (11.1%). Respondents reported “lack of advertising” (32.2%), “lack of trust in practitioner” (23.7%), “nonscientific” (23.0%), and “expensive cost” (21.1%) as the weaknesses of CAM.

The information about CAM was gained mainly from the mass media including print and broadcast media (40.1%), internet (37.6%), and family and friends (35.3%). Other CAM information resources that they reported were Western medicine hospitals (7.1%), Korean traditional medicine hospitals (6.6%), CAM practitioners (4.6%), and education including public lectures (3.9%).

## 4. Discussion

In this study, most common health condition in Korean young adults was musculoskeletal pain including neck, shoulder, and back pain. Korean young adults used CAM mainly to treat these musculoskeletal pains while they did exercise mainly to lose weight, promote health, and have a positive body image. Other reasons for CAM use were “chronic fatigue”, “stress management”, and “disease prevention and health promotion”.

Previous studies about CAM use in university students in the US, India, and the UK reported the similar results that reasons for CAM use were maintenance of physical and mental fitness [7], stress, maintenance of health, and nutrition [6]. Our results also indicate that compared to Korean older adults, Korean young adults presumably have different reasons for CAM use: A previous study reported that the most common reason for CAM use was “lack of energy” followed by “disease prevention” in Korean older adults (30-69 years) [18]. Nonetheless, our results were largely consistent with other adult studies as previous studies about CAM use among the general population in all over the world and the US reported that pain-related conditions including back and neck pain were the common health problems associated with CAM use [1, 19, 20].

The extent of belief in outcomes of health behavior seems to be related to commitment to behavior change: Beliefs in the importance of taking regular exercise for health maintenance were high in young adults and associated with exercise participation [15]. In this study, Korean young adults primarily perceived “relief of pain and symptoms” as the benefit of CAM and thus it seems that the perception of Korean young adults might be reflected in the reason for CAM use.

Exercise has been known to be effective for ameliorating symptoms and risks for diseases including obesity, cardiovascular disease, some cancers, diabetes, osteoporosis, anxiety, and depression [14, 24]. Accordingly, participation of exercise has been recommended to promote health and maintain a healthy weight [14]. Importantly, motives for participation in exercise are known to be influenced by age [25, 26]. Consistent with our results, previous studies reported that reasons for exercise in young adults in Europe, the US, and Australia were the management of weight and health and appearance while the important motive in older adults was health benefits [15, 25–28].

According to a previous survey in South Korea, visiting rate of Korean traditional medicine doctors increased between 2008 and 2011 from 45.8% to 69.3% of the respondents and the majority of visitors received the acupuncture treatment and moxibustion and cupping therapies [29]. Similarly, our results also showed that acupuncture was most frequently used therapy in young adults followed by massage, moxibustion and cupping therapies, and yoga. Meanwhile, according to the findings of previous studies, CAM treatments frequently used among university students in the US, India, and the UK were dietary supplements including vitamin and herbs, yoga, massage, meditation, and deep breathing exercises [6–8]. Previous studies about CAM use in the general adult population also showed that dietary supplements and herbs were the most frequently used CAM therapy throughout the world including South Korea (followed by Korean traditional medicine), Japan, Malaysia, the EU, and the US [3, 18, 30–32].

Our results regarding factors related to CAM use in young adults were consistent with the findings of previous studies: Sociodemographic factors such as sex (women), older age, higher income, higher level of education [1, 3], and poor health status [19, 33] were known to be as predictors of CAM use in older adults in all over the world, the US, and Europe. In the present study, higher prevalence of CAM use was observed in women compared to men and in 30-39 years of age group compared to 20-29 years of age group. Users had a higher level of education and were married and employed. Individuals who had any religion were more likely to use CAM than those without religion. In addition, young adults were more likely to use CAM when they had health problems. However, only education level, religious status, and presence of health problems were the independent factors associated with CAM use after controlling for other covariates.

This study has possible shortcomings including limitations of generalizability. The survey was conducted among Korean young adult members of fitness centers in order to compare the reasons for CAM use to those for exercise, but the study population may not accurately represent the general Korean young adult population. Nevertheless, Korean young adults may be unique and interesting for the study of the patterns and perceptions of CAM use compared with Korean older adults and those in Japan, China, and Western countries. First, in South Korea, unlike Japan and Western countries, a dual licensing system of medical doctors exists: a license for Western medical doctors and the other license for Korean traditional medicine doctors [29, 34]. Second, the government supports to promote Korean traditional medicine research since 2008 may have a considerable influence on the improvement in attitudes toward Korean traditional medicine in South Korean society [29]. These changes in attitude toward Korean traditional medicine and unique medical system of South Korea could influence on the patterns of CAM use in Korean young adults.

In conclusion, this study suggested that the main purposes of CAM and exercise are different in young adults in South Korea: They did exercise mainly to lose weight, to promote health, and to have a positive body image while they used CAM mainly to relieve musculoskeletal pains. Our results also showed that use of CAM was significantly associated with education level, religious status, and health status in young adult users of fitness centers. Notably, majority of respondents answered that they got CAM information from the mass media and the internet instead of CAM officials including Korean traditional medicine doctors and CAM practitioners, suggesting that official efforts to confirm the adequacy of the information may be needed to enhance the use of CAM in young generation. In the future, a comparison study among the same generations in different countries will be needed to fully understand patterns, reasons, and perspectives of exercise and CAM use in diverse cultural, social, and economic backgrounds.

## Figures and Tables

**Table 1:** Demographic characteristics and the prevalence of CAM use in lifetime.

Variables	Category	Total	CAM Users	Non-CAM Users
		(n = 649)	(n = 526)	(n = 123)
Age	20-29 years	358 (55.2)	280 (53.2)	78 (63.4)
	30-39 years	291 (44.8)	246 (46.8)	45 (36.6)
Sex	Male	289 (44.5)	224 (42.6)	65 (52.8)
	Female	360 (55.5)	302 (57.4)	58 (47.2)
Marital status	Single	572 (88.1)	457 (86.9)	115 (93.5)
	Married	77 (11.9)	69 (13.1)	8 (6.5)
Education	High school or less	166 (25.6)	120 (22.8)	46 (37.4)
	College or higher	483 (74.4)	406 (77.2)	77 (62.6)
Religious status	None	441 (68.0)	345 (65.6)	96 (78.0)
	Have a religion	208 (32.0)	181 (34.4)	27 (22.0)
Employment status	Unemployed	150 (23.1)	113 (21.5)	37 (30.1)
	Employed	499 (76.9)	413 (78.5)	86 (69.9)
Monthly family income	<2 million KRW	134 (20.6)	107 (20.3)	27 (22.0)
	2-4 million KRW	325 (50.1)	267 (50.8)	58 (47.1)
	4-6 million KRW	122 (18.8)	99 (18.8)	23 (18.7)
	≥6 million KRW	68 (10.5)	53 (10.1)	15 (12.2)
Self-perceived health status	Very poor/poor	96 (14.8)	85 (16.2)	11 (8.9)
	Fair	254 (39.1)	204 (38.8)	50 (40.7)
	Good/very good	299 (46.1)	237 (45.0)	62 (50.4)
Presence of health problems	No	371 (57.2)	277 (52.7)	94 (76.4)
	Yes	278 (42.8)	249 (47.3)	29 (23.6)
Ten most common problems^*∗*^				
Neck/shoulder pain		203 (31.3)		
Headache		72 (11.1)		
Back pain		69 (10.6)		
Sleep disorder		52 (8.0)		
Stomach disease		34 (5.2)		
Depression/anxiety/emotional problem		27 (4.2)		
Arthritis/ rheumatism		16 (2.5)		
Hypertension		16 (2.5)		
Hyperlipidemia		11 (1.7)		
Respiratory disease		10 (1.5)		
Average number of health problems		2.0 ± 1.2		

Data are presented as n (%) or mean ± standard deviation.

^*∗*^Multiple responses; respondents who have diseases and symptoms (n = 278).

**Table 2:** Type and satisfaction of CAM used in lifetime (n = 649).

Categories of CAM used^*∗*^	CAM Users^*∗*^	Satisfaction^*∗∗*^
	N (%)	Mean ± SD
*Acupuncture*	**423 (65.2)**	**3.42±1.09**
*Massage *	**313 (48.2)**	**4.03±1.16**
*Moxibustion and cupping*	**271 (41.8)**	**3.43±1.09**
*Yoga*	**240 (37.0)**	**3.63±1.23**
*Diet-based therapies*	**193 (29.7)**	3.37±1.33
*Pilates*	**166 (25.6)**	**3.57±1.36**
*Natural products (Ginseng)*	**156 (24.0)**	3.41±1.12
*Taping therapy*	**132 (20.3)**	**3.64±1.27**
*Aroma therapy*	**77 (11.9)**	**3.81±1.29**
*Natural products (Others)*	**72 (11.1)**	**3.56±1.12**
Spinal manipulation	71 (10.9)	**3.66±1.45**
Meditation	55 (8.5)	3.11±1.34
Natural products (Herbs)	50 (7.7)	3.02±1.38
Chuna (Korean manipulative therapy)	49 (7.6)	**3.67±1.48**
Hand acupuncture	43 (6.6)	2.98±1.35
Vitamin and mineral supplements	34 (5.2)	3.09±1.53
Folk medicine (Animal extracts, etc.)	27 (4.2)	2.67±1.75
Art therapy	20 (3.1)	3.30±1.84
Light therapy	18 (2.8)	2.94±1.83
Therapeutic spa in hot springs	17 (2.6)	2.94±1.85
Tai chi	14 (2.2)	1.79±1.93
Therapeutic prayer	13 (2.0)	2.00±2.08
Hypnosis	12 (1.8)	1.67±1.88
Ayurveda	11 (1.7)	1.45±1.70
Homeopathic treatment	11 (1.7)	2.09±2.07
Progressive relaxation	10 (1.5)	2.30±2.11
Kouksundo (Korean mind-body practice)	8 (1.2)	1.25±1.75
Magnetic therapy	8 (1.2)	1.13±1.64
Guided imagery	6 (0.9)	1.33±2.07
Qi gong	3 (0.5)	1.67±2.89

Any CAM use in lifetime	526 (81.0)	

Number of CAM therapies used		
1-3	220 (41.8)	
4-6	182 (34.6)	
≥7	124 (23.6)	
Average number of therapies used	4.8 ± 3.5	

Data are presented as n (%) or mean ± standard deviation (SD).

^*∗*^Multiple responses; ten most commonly used CAM therapies are marked in bold.

^*∗∗*^Ten most satisfied CAM therapies are marked in bold.

**Table 3:** Predictors of CAM use in lifetime (n = 649).

Variables	Category	Unadjusted OR^*∗*^	*P*	Adjusted OR^*∗*^	*P*
		(95% CI)	value	(95% CI)	value
Age	20-29 years	Reference		Reference	
	30-39 years	**1.52 (1.02-2.28)**	0.042	1.11 (0.70-1.77)	0.649
Sex	Male	Reference		Reference	
	Female	**1.51 (1.02-2.24)**	0.040	1.42 (0.94-2.17)	0.099
Marital status	Single	Reference		Reference	
	Married	**2.17 (1.02-4.64)**	0.046	2.05 (0.93-4.56)	0.077
Education	High school or less	Reference		Reference	
	College or higher	**2.02 (1.33-3.07)**	0.001	**1.76 (1.11-2.77)**	0.015
Religious status	None	Reference		Reference	
	Having a religion	**1.87 (1.17-2.97)**	0.008	**1.75 (1.08-2.83)**	0.022
Employment status	Unemployed	Reference		Reference	
	Employed	**1.57 (1.02-2.44)**	0.043	1.46 (0.88-2.43)	0.142
Monthly family income	<2 million KRW	Reference		Reference	
	2-4 million KRW	1.16 (0.70-1.93)	0.564	1.00 (0.57-1.73)	0.990
	4-6 million KRW	1.09 (0.58-2.02)	0.794	0.90 (0.46-1.75)	0.756
	≥6 million KRW	0.89 (0.44-1.82)	0.752	0.96 (0.46-2.04)	0.921
Presence of health problems	No	Reference		Reference	
	Yes	**2.91 (1.86-4.57)**	0.000	**2.79 (1.72-4.54)**	0.000
Self-perceived health status	Very good/good	Reference		Reference	
	Fair	1.07 (0.70-1.62)	0.759	0.88 (0.56-1.37)	0.565
	Poor/very poor	**2.02 (1.02-4.02)**	0.045	1.31 (0.62-2.75)	0.475

^*∗*^Significant odds ratios (ORs) are marked in bold; CI, confidence interval.

**Table 4:** Reasons for CAM use and doing exercise (n = 649).

Variables	N	%
*Top ten reasons for CAM use* ^*∗*^		
Neck/shoulder pain	211	32.5
Back pain	79	12.2
Chronic fatigue	50	7.7
Stress management	50	7.7
Arthritis	37	5.7
Disease prevention and health promotion	33	5.1
Obesity management	30	4.6
Headache	25	3.9
Stomach disease	16	2.5
Insomnia	11	1.7
*Reasons for exercise* ^*∗∗*^		
Weight loss	408	62.9
Health promotion	349	53.8
To have a positive body image	291	44.8
Stress management	163	25.1
Leisure activity	121	18.6
Relief of pain	56	8.6
Disease treatment	28	4.3
Others	5	0.8

^*∗*^Multiple responses; average number of choices was 2.1 ± 1.2.

^*∗∗*^Multiple responses; average number of choices was 2.2 ± 1.1.

**Table 5:** Perceived benefits and weakness of CAM and sources of CAM information.

Variables	N	%
*Benefits of CAM* (N=647)^*∗*^		
Relief of pain and symptoms	376	57.9
Psychological and emotional wellbeing	260	40.1
Promotion of health and physical fitness	215	33.1
Improvement of immune function	79	12.2
Treatment of disease	72	11.1
*Weaknesses of CAM* (N = 649)		
Lack of advertising	209	32.2
Lack of trust in practitioner	154	23.7
Non-scientific	149	23.0
Expensive cost	137	21.1
*Sources of CAM information* (N = 649)^*∗∗*^		
Mass media including print and broadcast media	260	40.1
Internet	244	37.6
Family and friends	229	35.3
Western medicine hospitals	46	7.1
Korean traditional medicine hospitals	43	6.6
CAM practitioners	30	4.6
Education including public lectures	25	3.9
Others	42	6.5

^*∗*^Multiple responses; average number of choices was 1.5 ± 0.8.

^*∗∗*^Multiple responses; average number of choices was 1.4 ± 0.7.

## Data Availability

The data used to support the findings of this study are available from the corresponding author upon request.
